# Verb bias and verb-specific competition effects on sentence production

**DOI:** 10.1371/journal.pone.0180580

**Published:** 2017-07-03

**Authors:** Malathi Thothathiri, Daniel G. Evans, Sonali Poudel

**Affiliations:** Department of Speech, Language and Hearing Sciences, The George Washington University, Washington, D.C., United States of America; Birkbeck College, UNITED KINGDOM

## Abstract

How do speakers choose between structural options for expressing a given meaning? Overall preference for some structures over others as well as prior statistical association between specific verbs and sentence structures (“verb bias”) are known to broadly influence language use. However, the effects of prior statistical experience on the planning and execution of utterances and the mechanisms that facilitate structural choice for verbs with different biases have not been fully explored. In this study, we manipulated verb bias for English double-object (DO) and prepositional-object (PO) dative structures: some verbs appeared solely in the DO structure (DO-only), others solely in PO (PO-only) and yet others equally in both (Equi). Structural choices during subsequent free-choice sentence production revealed the expected dispreference for DO overall but critically also a reliable linear trend in DO production that was consistent with verb bias (DO-only > Equi > PO-only). Going beyond the general verb bias effect, three results suggested that Equi verbs, which were associated equally with the two structures, engendered verb-specific competition and required additional resources for choosing the dispreferred DO structure. First, DO production with Equi verbs but not the other verbs correlated with participants’ inhibition ability. Second, utterance duration prior to the choice of a DO structure showed a quadratic trend (DO-only < Equi > PO-only) with the longest durations for Equi verbs. Third, eye movements consistent with reimagining the event also showed a quadratic trend (DO-only < Equi > PO-only) prior to choosing DO, suggesting that participants used such recall particularly for Equi verbs. Together, these analyses of structural choices, utterance durations, eye movements and individual differences in executive functions shed light on the effects of verb bias and verb-specific competition on sentence production and the role of different executive functions in choosing between sentence structures.

## Introduction

Language contains different structural options for expressing meaning. For example, a transfer event could be described using either a double-object (DO) or a prepositional-object (PO) dative structure (e.g., DO: *Ayesha gave John the book*; PO: *Ayesha gave the book to John*). How do speakers choose between these options? Verb bias—the frequency with which verbs appear in different structures—broadly influences language comprehension and production [1–11]. Thus, prior language experience is known to influence subsequent language use. However, the effect of prior statistical experience on how speakers encode events and plan and execute utterances, and the mechanisms by which they choose between different structural options have not been fully explored. In this study, we manipulated verb bias for English dative constructions in a lab-based training protocol and investigated the effects of that training on subsequent naturalistic sentence production. Additionally, we monitored speakers’ eye movements and measured utterance characteristics and executive functions to more fully understand how prior experience influences subsequent production and identify the mechanisms that could support structural choices.

Previous studies have shown that verb bias influences structural choices during language production. For example, whether participants include or omit “that” when producing a sentence with an embedded clause varies according to how often the verb generally appears with embedded clauses ([2]. See also [3,12]). Verbs that appear more frequently in structures where the verb and its complement are non-adjacent are more likely to allow the shifting of complements to different sentence positions [13]. Verb-specific syntactic probabilities can also affect pronunciation—words in an utterance tend to be shortened when verbs appear in highly likely syntactic structures and lengthened when they appear in unlikely structures [14].

While studies such as those described above demonstrate that statistical biases present naturally in language can affect language use, they cannot fully distinguish the effects of syntactic probabilities from semantic factors that condition how different verbs are used. Other studies have circumvented this constraint on interpretation by manipulating verb bias within lab-based training sessions, using an artificial language with new sentence structures [8–10], a natural language with new verbs [15], or a natural language with familiar verbs but experimentally manipulated verb-structure associations [1,16]. In these paradigms, verbs were randomly assigned to different bias conditions, thereby enabling separation of verb bias from verb semantics. The results broadly show that people are more likely to use verbs in a statistically associated structure than in an unassociated structure during spoken as well as written production.

The current study used lab-based training and random assignment of verbs to different bias conditions as described above. Participants were trained with dative verbs that appeared only in DO, only in PO, or equally in both (hereafter called DO-only, PO-only and Equi verbs, respectively). Subsequent to training, they generated new dative sentences to describe transfer events not seen before. We evaluated whether the speakers’ structural choices varied systematically according to verb bias. Additionally, we tested hypotheses about the role of different executive functions in making structural choices and the effect of prior statistical experience on how speakers encoded and described the events.

Executive function is an umbrella term for several regulatory abilities. These abilities facilitate goal-directed behavior via maintenance of current goals, inhibition of competing information, switching between contexts, and other related functions. We hypothesized that two specific executive functions might be relevant for making structural choices. When multiple structural options are available for expressing the same meaning, inhibition of one option might facilitate production of the other option. For example, during the process of describing a transfer action, speakers could activate both DO and PO dative structures and inhibit PO to select DO (which is generally dispreferred relative to the PO in English, see e.g., [17]). Alternatively, speakers may choose between different structures by switching between different perspectives or rules for describing an event. For example, they might encode the event as being more about the recipient than the theme leading to the use of the DO structure (*Ayesha gave* ***John*** *something*) or being more about the theme than the recipient leading to the use of the PO structure (Ayesha gave ***the book*** to someone). A role for switching in language production has been hypothesized previously for switching between different languages in bilinguals [18,19]. Here, we examined whether this ability might be relevant for choosing between structures within a single language (see more below).

Exploration of individual differences in different executive function tasks suggests at least a partial dissociation between switching and other functions such as inhibition [20,21]. Specifically, switching appears to involve flexibility in using different task rules in different contexts, which is distinct from the ability to inhibit distracting information [20,21]. Consistent with such a dissociation and directly related to sentence production, a previous study found that structural choice in different verb bias conditions in an artificial language correlated differently with inhibition and switching performance [9]. In that study, inhibition was measured using the Stroop task, where participants were asked to name the font color of written color words. Critical trials required the inhibition of an automatic reading response in order to name the visual color. Switching was measured using the Number-Letter task. Participants viewed number-letter combination stimuli (e.g., “7G”) and were asked to respond according to a number rule (Is the number odd or even?) or a letter rule (Is the letter a consonant or a vowel?). Critical trials required switching between the two rules. Interestingly, the results suggested that the executive functions used for choosing between structures might be different for different verb bias conditions. Stroop performance correlated with the production of a dispreferred structure with Equi verbs, which were associated equally with two structures. In contrast, Number-Letter performance correlated with the production of a dispreferred structure with single-structure verbs, which were consistently associated with only one structure. Coupled with neuroimaging evidence for the involvement of different brain networks for different verb bias conditions, these results suggested that the *same* structural output might be supported by *different* underlying mechanisms for different verbs [9]. In particular, verbs that appear equally often in two competing structures and automatically activate both structures might require additional resources such as the inhibition of one structure to facilitate production of the other. Accordingly, in the present study, we examined whether Stroop inhibition performance was correlated more reliably with DO production in the Equi bias condition than in the DO-only or PO-only conditions. It is worth noting here that this *empirically derived* prediction might contradict some a priori intuitions. We return to this issue in Discussion.

The availability of multiple structural options can affect not only the ultimate choice of sentence structure but also the time course of planning and executing the utterance. Two theories proposed in the literature make contrasting predictions with respect to whether this factor will speed up or slow down sentence production. Under the *incremental model*, flexibility in positioning different noun arguments is predicted to facilitate sentence production [22]. Thus, depending on whether the recipient noun or the theme noun is more accessible on any given trial, the production system can more easily choose between the DO structure (place the recipient noun phrase first after the verb) or the PO structure (place the theme noun phrase first) if both options are available. In contrast, the alternative *competitive model* makes the opposite prediction: that the availability of multiple structures will induce competition and slow down sentence production. The weight of the current evidence is tilted towards the latter model [23,24]. For example, Korean speakers were slower to begin an utterance when the nouns provided to them were not case-marked and allowed for multiple structural options than when the nouns were case-marked and only one structure was viable [23]. Taking a cross-language approach, Myachykov and colleagues found that formulating a canonical active voice structure took longer in Russian than in English. They argued that this was due to the greater number of structures available in Russian than English [24]. In the present study, the availability of structural options may be conceptualized in terms of verb bias. Equi verbs, by virtue of their equal association with both structures, should be more likely to activate both structural options than DO-only or PO-only verbs. Consistent with this idea, one previous study found that Dutch dative verbs that were not significantly biased towards DO or PO but appeared roughly equally in both—i.e., Equi verbs in our terminology—activated more structural options during sentence formulation than verbs strongly biased towards either DO or PO [25]. Thus, competition effects can arise not only at the language-wide level based on the availability of structural alternatives but also at a verb-specific level based on prior statistical associations. We refer to the latter hereafter as *verb-specific competition*. Synthesizing the findings from the previous studies, we hypothesized and tested whether sentence production with Equi verbs would generate verb-specific competition and lead to delays relative to single-structure verbs in speech onset latencies and/or durations prior to the choice of a DO or a PO dative. In addition, we conducted exploratory analyses of participants’ eye movements during the video and the response phases to evaluate whether prior statistical experience influenced how participants encoded the events or planned their utterances. Together, our results shed light on how verb bias and verb-specific competition influence sentence production and highlight the potential role of different executive functions in making structural choices.

## Method

### Participants

Ninety-five right-handed, native English speakers completed the study. Seven were excluded because they did not pass our pre-established criterion of producing at least one each of DO and PO structures. The final list included eighty-eight participants (18–36 years; Mean age = 20.2; 60 female). Each participant self-reported handedness, language history, and color vision ability. This study was reviewed and approved beforehand by the Institutional Review Board at The George Washington University. All participants gave consent under the approved protocol.

### Materials and procedure

Participants completed four relevant tasks: Training, Production Test, Stroop, and Number-Letter (Note: We have omitted discussion of a fifth task, which tested participants’ memory for the training sentences, because it did not yield clear results and is not relevant to the questions at hand).

#### Training

**Fig 1:**
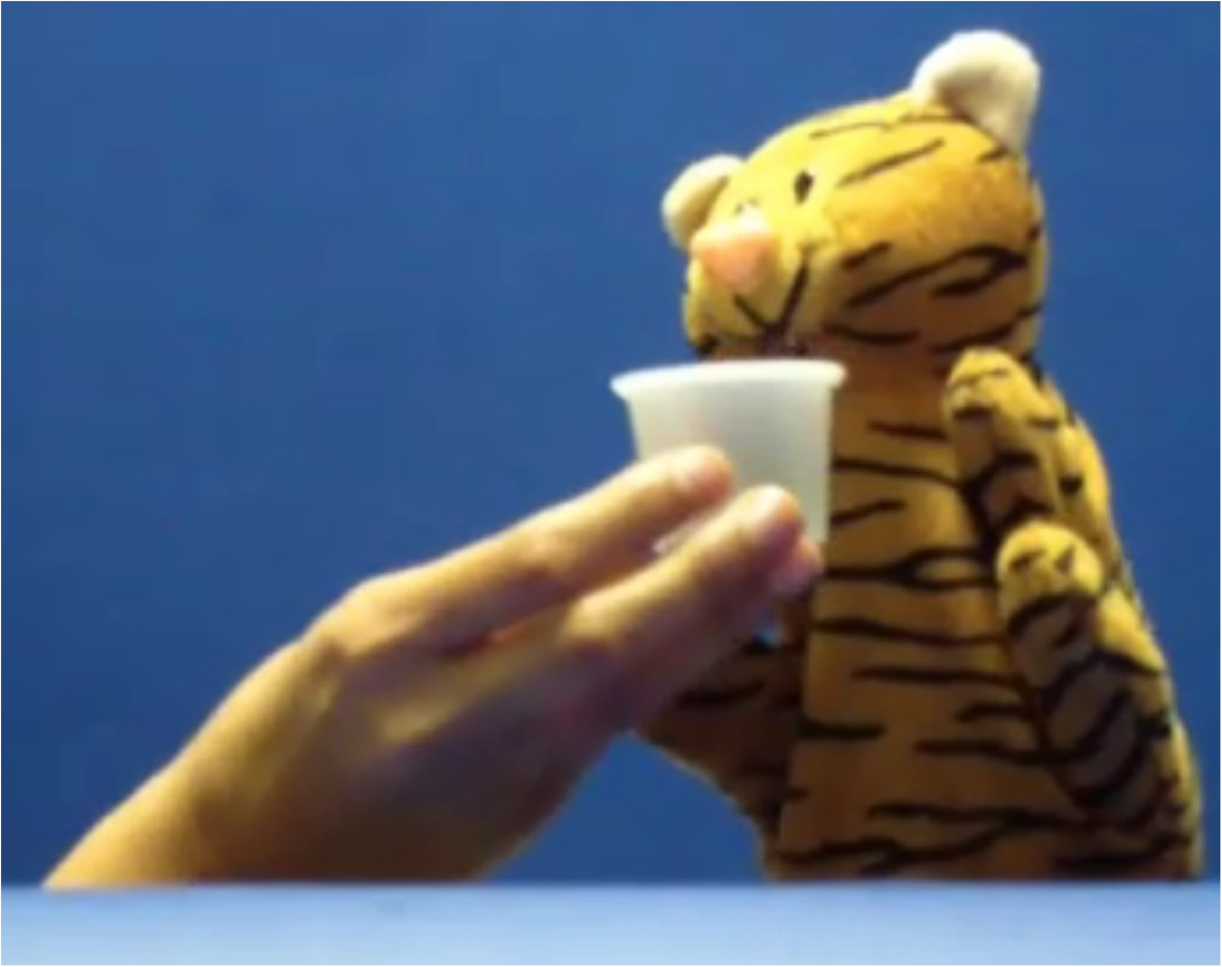
Example training video. Participants saw a transfer event, and heard and repeated a DO or a PO description (DO: *Kate gave the tiger the cup*; PO: *Kate gave the cup to the tiger*).

During training, participants watched 120 videos of transfer actions, heard accompanying dative sentences, and repeated the sentences. Stimuli were presented using E-prime. The videos depicted a human hand transferring an object to a puppet animal (Fig 1). Six animals (donkey, giraffe, lion, monkey, tiger, zebra) and six objects (apple, candle, cup, flower, fork, and hat) appeared 20 times each. The accompanying auditory sentences contained ten dative verbs (bring, give, mail, offer, pass, roll, show, slide, throw, toss), each of which appeared 12 times. Of the ten verbs, four were presented only in DO (e.g., *Kate gave the tiger the cup*), four only in PO (e.g., *Kate passed the flower to the zebra*), and two equally in both structures (Equi). Each verb appeared in each bias condition across five lists. Participants were randomly assigned to a list. Sentences consisted of all possible combinations of verbs and nouns except that we avoided phonological overlap (e.g. toss and tiger did not appear together). Trial order was pseudorandomized such that verbs did not repeat consecutively, and there were no more than three trials from the same verb bias condition in a row. At the end of a trial, participants pressed a key to proceed to the next trial.

#### Production test

Following training, participants completed a production test, where they watched and then freely described new videos. The test videos contained different animals (bear, cat, cow, dog, frog, pig) and objects (feather, key, knife, mitten, napkin, ring) than training. This ensured that participants generated new sentences and did not rely on memory. Each verb appeared four times, and each animal/object appeared six or seven times, in a total of 40 test trials. As before, we avoided phonological overlap (e.g. mail and mitten). Trial order was pseudorandomized such that consecutive trials did not contain the same verb or nouns. There were no more than two trials from the same verb bias condition in a row. Each trial presented a written verb, a video, and then a slide with a green circle when participants were instructed to speak. Participants were given a maximum of four seconds for their verbal response. When done, they pressed a key to proceed to the next trial. We tracked participants’ eye movements during the production test with an EyeLink 1000 Plus desktop mounted eyetracker. Samples were recorded at a rate of 1000Hz with a spatial resolution of approximately 0.5°. Test stimuli were presented using the EyeLink compatible software, Experiment Builder.

#### Stroop

In the Stroop task, participants responded to the ink color of different words using color-coded keys (blue, green, yellow). Stimuli were neutral (e.g., *horse* in blue ink), congruent (e.g., *blue* in blue ink), incongruent-eligible (e.g., *green* in blue ink), or incongruent-ineligible (e.g., *red* in blue ink). After a short practice (eight trials), participants completed 288 test trials (72 per condition) split into four blocks. Within each condition, each word appeared an equal number of times (Neutral: deal, farmer, horse, plenty, stage, tax; Incongruent-ineligibile: brown, orange, red; Congruent and Incongruent-eligible: blue, green, yellow). Each response was required 96 times. Order was pseudorandomized such that no word or response appeared more than three times in a row.

#### Number-Letter

In the Number-Letter task, participants saw a number-letter combination (e.g. 4E) in one of four quadrants. If the stimulus appeared in a top quadrant, they responded to the number by pressing 1 for odd and 2 for even. If the stimulus appeared in a bottom quadrant, they responded to the letter by pressing 1 for consonant and 2 for vowel. Stimuli consisted of eight letters (*M*,*R*,*G*,*K*,*A*,*E*,*I*,*U*) and eight numbers (*3*,*5*,*7*,*9*,*2*,*4*,*6*,*8*). Participants first completed a block each of the Number and Letter tasks separately (32 trials per block). Subsequently they practiced intermixing the two tasks (10 trials) and then completed the critical test block (128 trials). In the critical block, the stimulus rotated clockwise through the four quadrants such that the task alternately stayed the same or switched. Number of trials involving each stimulus and response was counterbalanced. Order of stimuli was pseudorandomized such that no stimulus appeared more than twice in a row and neither of the two responses was required more than four times in a row.

### Analysis

#### Production test

We transcribed and coded participants’ spoken descriptions of the test videos. Only responses containing the correct verb, correct nouns (or near synonyms e.g., glove for mitten), and a complete dative structure including an agent, a recipient and a theme were accepted as DO/PO. A majority of the accepted responses (89.5%) were the expected DO and PO sentences (DO: *Kate gave the <animal> the <object>*; PO: *Kate gave the <object> to the <animal>*). Other accepted responses also contained the dative structure but with modifications such as using a pronoun for the agent (*She gave…*), another preposition (e.g., *over to* instead of *to*), elaborations (e.g., *Kate proceeded to give*. . .), alternate inflections (e.g., *Kate gives…*), and passivizations (e.g., *The cow was tossed the feather*). Non-dative structures and errors (e.g., repeats, restarts, incorrect verb, incorrect noun) were coded as Other. Response type (DO or PO) was analyzed using mixed effects logistic regression (glmer function with the bobyqa optimizer. lme4 version 1.1.12 in R). The model contained the fixed effect of verb bias coded as an orthogonal polynomial contrast, and random intercepts and slopes by participant and item.

For the utterance analysis, we measured onset latencies, and durations prior to the choice of a DO/PO structure (*Kate gave the…* Hereafter called pre-choice duration) using Audacity. Trials where latencies were more than 3 standard deviations from the mean for each participant were excluded (1.7%). Only correct trials with the expected words and structures were analyzed. The mixed effects linear regression models (lmer function in R) contained fixed effects of verb bias (coded as a polynomial contrast), response type (DO/PO) and the interaction, and random intercepts and slopes by participant and item. Models containing the full set of random effects did not converge. Therefore, we simplified the random effects structure in a stepwise fashion (leaving the fixed effects structure intact). The final models contained random by-participant and by-item intercepts, by-participant slopes for verb bias, response type and the interaction, and by-item slopes for verb bias and response type.

To explore whether prior statistical experience influenced how participants encoded the events and/or planned and executed their utterances, we analyzed eye movements during the video as well as the response phase. Videos of the transfer actions proceeded from left to right (the agent on the left transferring the theme to the recipient on the right). Each video contained three interest areas: left, middle and right. While the theme moved between the different interest areas, the recipient stayed in the right interest area throughout the video. We analyzed looks to the right interest area prior to the theme getting there, in order to determine whether participants “looked ahead” to the recipient. Such a pattern could underlie accessing the recipient noun and planning a DO utterance. On each trial, we computed the proportion of time that a participant was looking at the right interest area during the interval from the onset of the video to the time at which the theme reached the recipient. For actions where the theme never physically reached the recipient (e.g., showing an object to an animal), we defined the interval using the average duration from all other videos. Proportions were empirical-logit transformed before analysis [26]. The mixed effects linear regression model contained the fixed effects of verb bias (coded as a polynomial contrast), response type and the interaction plus random intercepts and random slopes of verb bias and response type by participant and item. To summarize, in the video phase, we examined whether participants looked ahead to the recipient prior to the completion of the transfer action, and whether the likelihood of such looks varied according to verb bias and/or the eventual structure produced.

During the response phase, the only visual stimulus was a green circle centered on the screen. Thus, participants were generally expected to transition away from looking to the right interest area at the end of the video to looking at the middle during the response phase. However, we considered the possibility that participants might mentally replay the event when planning and executing the utterance and that this might be reflected in their looks to the right interest area (where the transfer action was completed in the video). Therefore, we conducted mixed effects regression analyses over empirical-logit transformed proportions of looks to the right interest area, as described above, also during the response phase. Specifically, we examined whether the proportions of looks varied according to verb bias and/or response type in the pre-choice interval following utterance onset and before the choice of DO/PO (*Kate gave the…*). The model contained the fixed effects of verb bias (coded as a polynomial contrast), response type and the interaction plus random intercepts by participant and item, random slopes for verb bias, response type and the interaction by participant, and random slopes for verb bias and response type by item.

#### Stroop and Number-Letter

For Stroop, the critical contrast was that between the incongruent-ineligible and the neutral baseline trials because the former trial type requires the inhibition of a competing representation (e.g., for *red* in blue ink, the representation corresponding to “red” must be inhibited). For each participant, we computed a normalized inhibition score as: (Mean accuracy on incongruent-ineligible trials–Mean accuracy on neutral trials)/(Mean accuracy on neutral trials) * 100. Higher scores indicate greater ability to inhibit a competing representation. For Number-Letter, the critical contrast was between trials that required participants to switch between number and letter rules and those where the rule stayed the same. For each participant, we computed a normalized switching score as: (Mean accuracy on switch trials–Mean accuracy on same trials)/(Mean accuracy on same trials) * 100. Higher scores indicate greater switching ability. For both tasks, we scaled the scores based on performance in a baseline comparison condition in order to diminish the contribution of individual differences in factors that were not of interest (e.g., threshold for responding). Using unscaled difference scores (Mean on critical trials minus Mean on baseline trials) yielded a similar pattern of results except in one case, which is described below. For both tasks, we computed accuracy rather than reaction time scores because we were interested in inhibitory/switching ability rather than efficiency. Because our free-choice language production task was self-paced, we reasoned that the ability to employ executive function—given sufficient time—was more relevant than speed-based measures (see also [9]). To assess the relationship between language production and executive function, we computed skipped Pearson correlations [27] between the above measures and percent DO produced in each condition. This robust regression analysis reduces the risk of false positives and negatives and is more appropriate when the data are contaminated with outliers and do not meet the assumptions of standard regression analyses (as in the present case). It detects and deletes bivariate outliers and adjusts for deleted data points when testing for significance [27]. Significant correlations are those with a confidence interval not spanning zero. As an added precaution against false positives, we corrected the p values corresponding to the correlations for family-wise error (p.adjust function in R, method = “fdr”, α = .05).

## Results

### Effect of verb bias on structural choices

**Fig 2:**
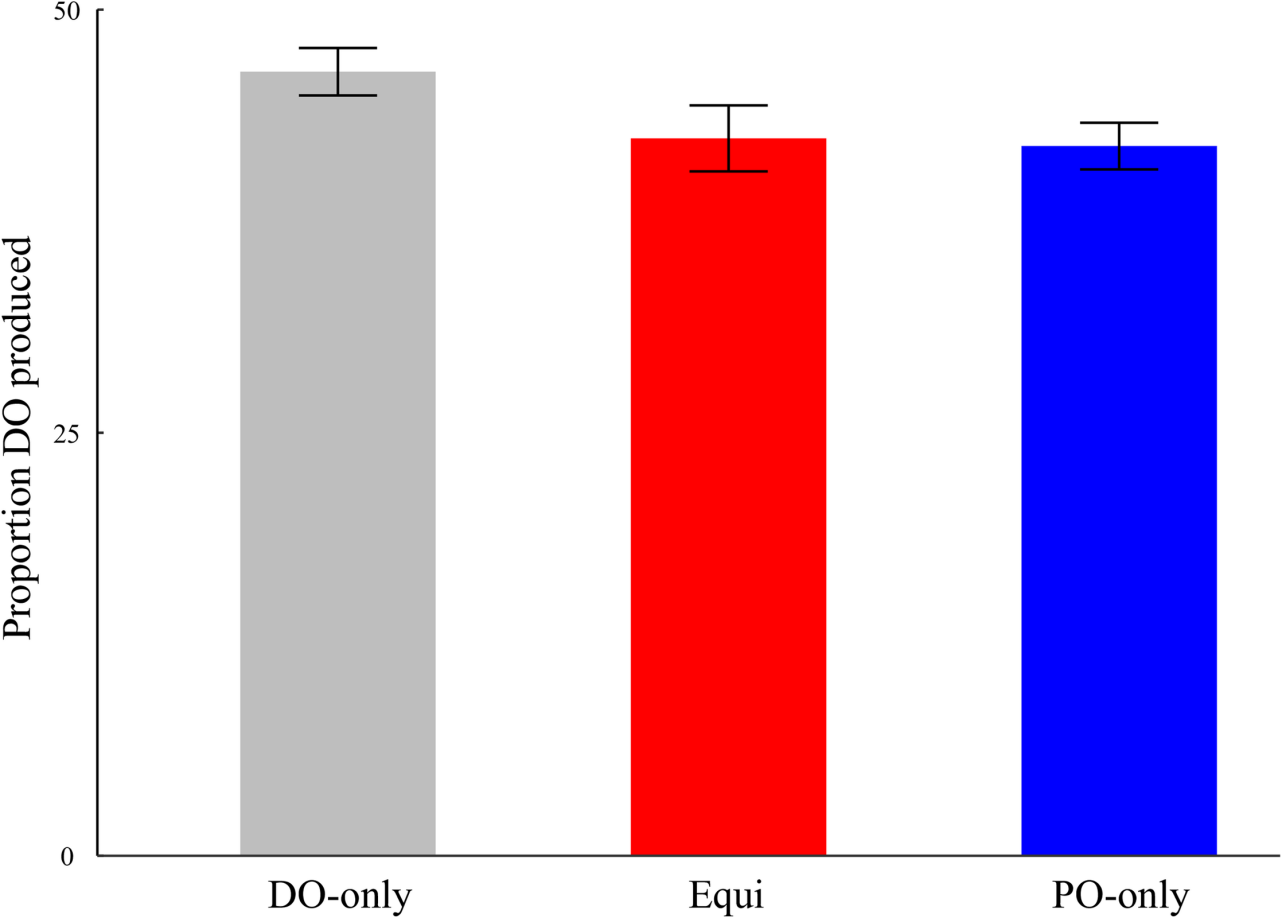
Proportion of DO responses produced in different verb bias conditions. Error bars denote standard errors.

Overall response percentages were 50.9% PO, 39.7% DO, and 9.4% other. Mixed effects modeling revealed a significant linear trend (DO-only > Equi > PO-only) in the likelihood of DO responses (Table 1). For visualization, the mean DO proportions (DO/(DO+PO) *100) produced in different conditions are shown in Fig 2. The observed pattern is consistent with an expected dispreference for DO overall (proportion<50%) combined with modulation according to verb bias (linear trend).

**Table 1:** Mixed model of the likelihood of a DO response in different verb bias conditions.

	Estimate	S.E.	Wald Z	p
*Fixed effects*				
Intercept	-.34	.16	-2.12	.033
Bias linear trend	-.15	.07	-2.03	.042
Bias quadratic trend	.05	.09	.56	.573

### Correlations between structural choices and executive function measures

**Fig 3:**
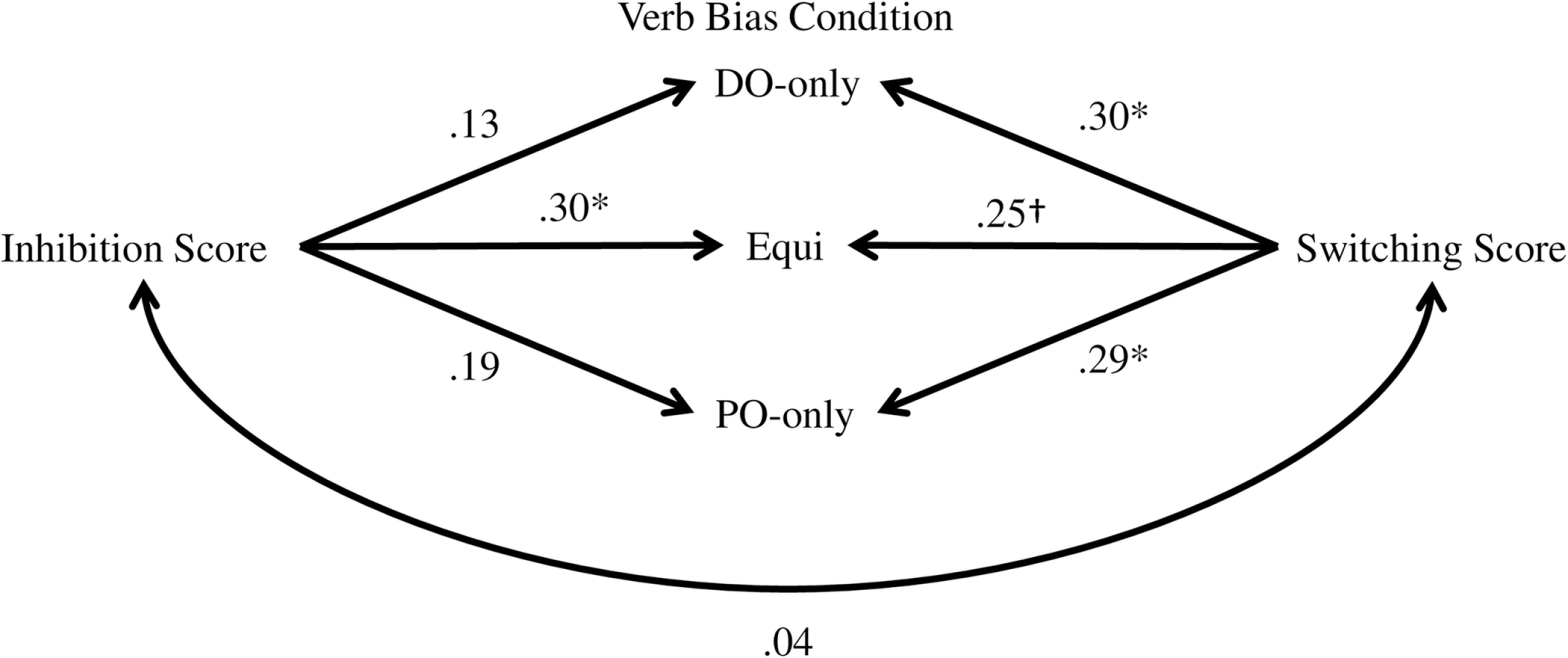
Correlations between DO production in different verb bias conditions and normalized inhibition and switching accuracy scores. * indicates significant correlation using either normalized or unscaled difference scores, † indicates significant correlation using normalized measures only.

Correlational analyses between structural choices and executive function measures revealed different patterns for different verb bias conditions (Fig 3). Switching scores correlated with proportion of DO produced in all three bias conditions (DO-only: r = .30 CI = [.12 .47]; Equi: r = .25 CI = [.05 .42]; PO-only: r = .29 CI = [.14 .45]). By contrast, inhibition scores correlated with proportion of DO produced with Equi verbs (r = .30 CI = [.15 .46]) but not DO-only (r = .13 CI = [-.06 .29]) or PO-only (r = .19 CI = [-.003 .38]) verbs. The robust correlations (between switching and production in all bias conditions and between inhibition and production in the Equi condition) were significant after correcting for multiple correlational tests (N = 6. See Methods). Using unscaled difference scores yielded a similar pattern, with the sole exception being that switching scores did not correlate with proportion of DO produced with Equi verbs (r = 0.19 CI = [-0.02 0.39]). Together, these results suggest that switching ability broadly facilitated the production of the dispreferred DO structure (potentially more robustly for DO-only and PO-only than for Equi verbs), and that inhibition ability might have more specifically facilitated DO production with Equi verbs. We tested the latter possibility directly by evaluating whether proportion of DO for Equi verbs showed a higher correlation with inhibition than that for DO-only and PO-only verbs (paired.r function in R). This comparison was statistically significant for Equi versus DO-only (t = 2.76, one-tailed p < .001) and marginally significant for Equi versus PO-only (t = 1.6, one-tailed p = .06).

### Utterance characteristics

**Fig 4:**
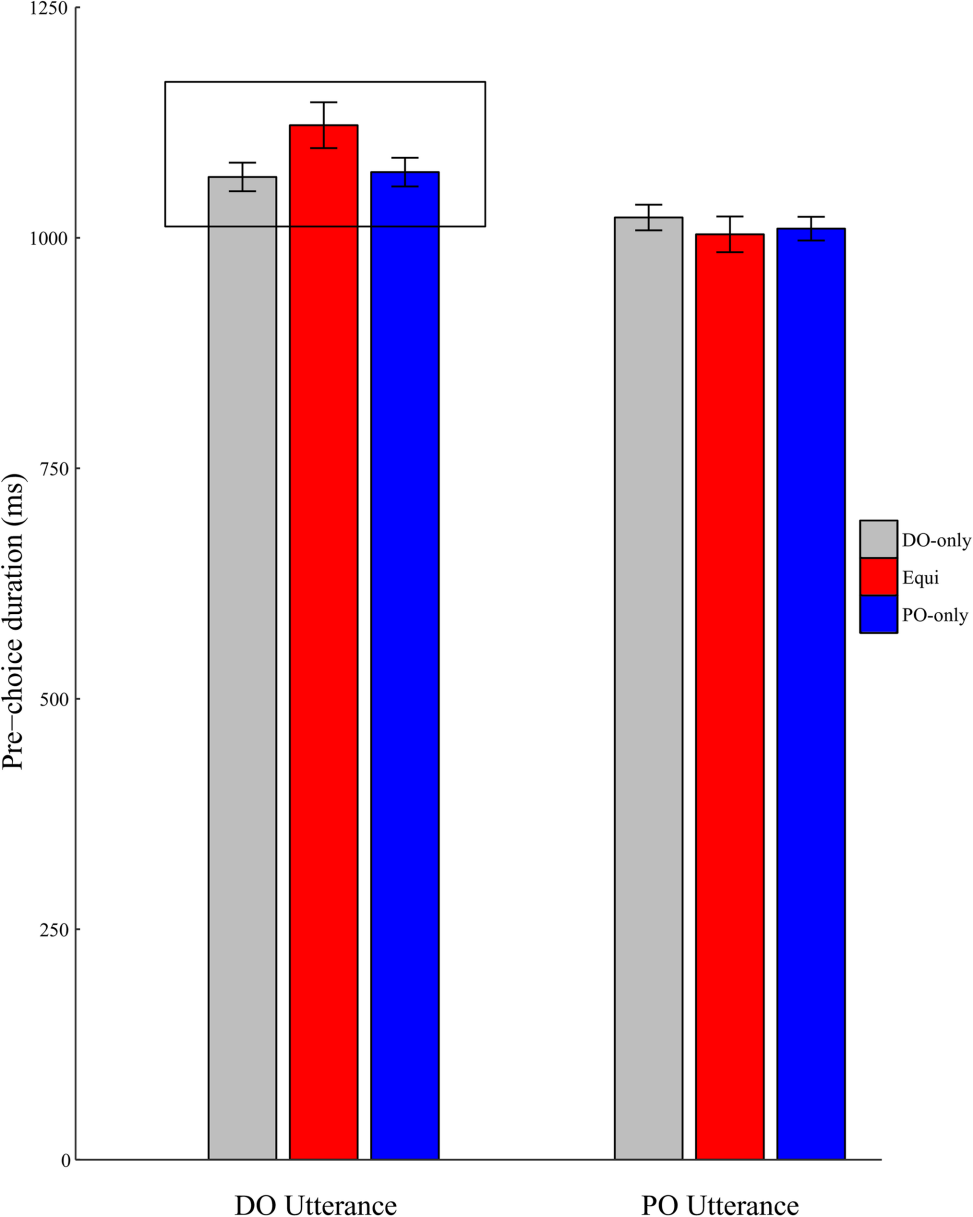
Pre-choice durations in different verb bias and response type conditions. Error bars denote standard errors.

Analysis of speech onset latencies did not reveal any significant effects of verb bias, response type or an interaction (p’s>.05). Thus, how quickly participants began an utterance did not differ reliably based on prior statistical experience or whether the produced structure was DO or PO. However, these factors led to a significant interaction in the analysis of pre-choice durations, i.e, in the time taken after the utterance began to when the choice between DO/PO was evident (Table 2). Specifically, there was an interaction between response type and a quadratic trend based on verb bias. For DO utterances, there was a significant quadratic DO-only < Equi > PO-only trend (Estimate = -41.86, S.E. = 17.01, t(248.10) = -2.46, p < .05). Equi verbs showed longer pre-choice durations than the other two verb types (M_DO-Only_ = 1066 ms, M_Equi_ = 1122 ms, M_PO-Only_ = 1071 ms) when the structure produced was DO. This pattern was not seen for PO utterances, which showed no quadratic trend (Estimate = 4.15, S.E. = 14.08, t(72.80) = .30, p>.7). Pre-choice durations for Equi verbs were not longer than for DO-only or PO-only verbs when the structure produced was PO (M_DO-Only_ = 1022 ms, M_Equi_ = 1004 ms, M_PO-Only_ = 1010 ms). The different patterns seen for DO and PO utterances are shown in Fig 4.

**Table 2:** Mixed model analysis of pre-choice durations.

	Estimate	S.E.	df	t	p
*Fixed effects*					
Intercept	1044.79	30.36	74.69	34.41	< .001
Response type	51.97	22.60	68.02	2.30	.025
Bias linear trend	.20	12.90	42.12	.02	.988
Bias quadratic trend	3.69	15.47	60.24	.24	.812
Response type * Bias linear trend	-11.32	17.80	103.70	-.64	.526
Response type * Bias quadratic trend	-50.19	24.94	101.17	-2.01	.047

### Eye movements

Eye movements during the video phase revealed a significant effect of response type (Estimate = -.45, S.E. = .13, t(99.60) = -3.56, p < .001). Proportion of looks to the right interest area was larger on trials where speakers eventually produced the DO structure compared to the PO structure. This suggests that attention to the recipient in the right interest area increased the likelihood of a DO response. Importantly, there was no effect of verb bias or interaction between verb bias and response type (p’s>.05) Thus, prior statistical experience did not appear to modulate how participants visually encoded the events.

**Fig 5:**
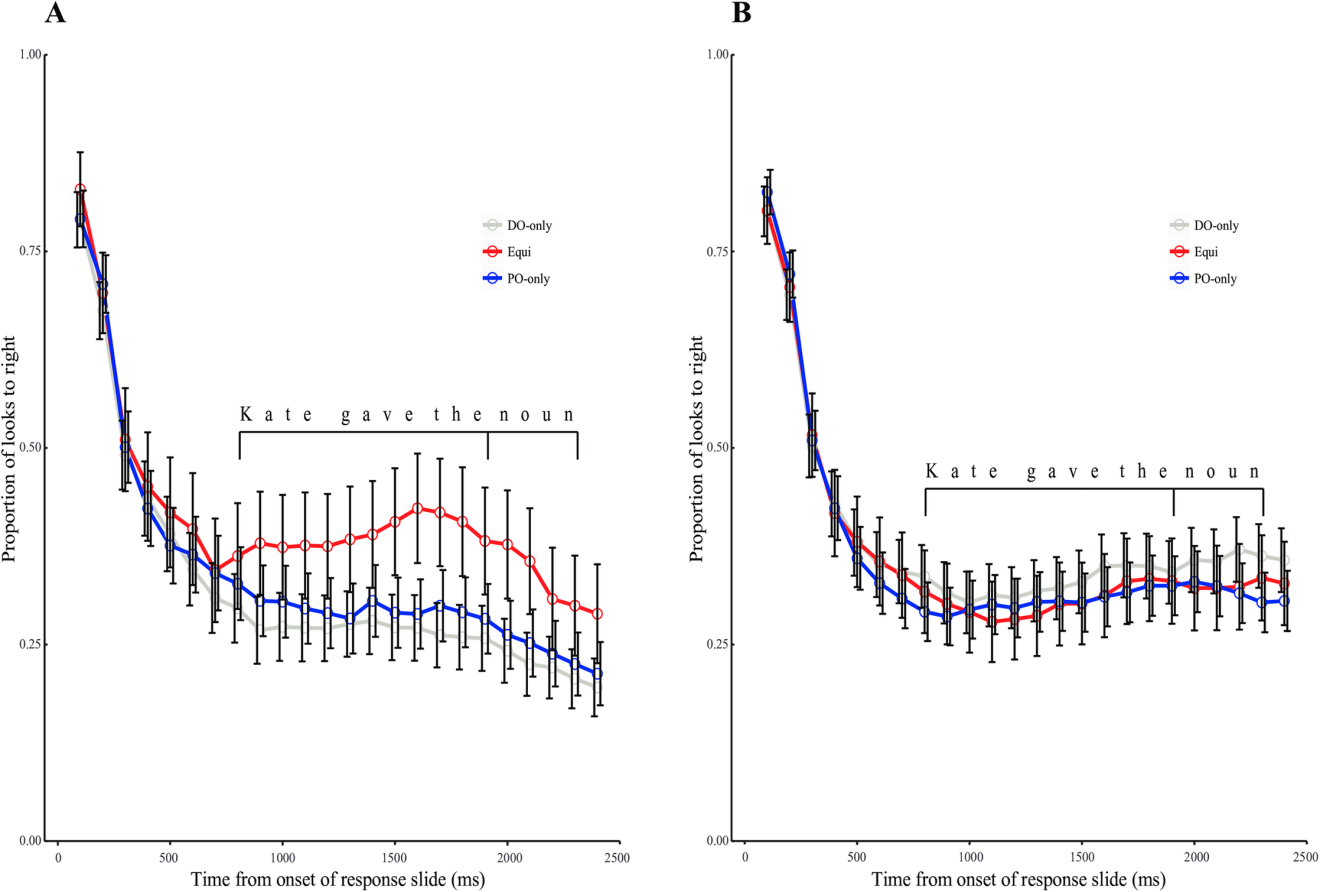
Eye movements during the response phase when participants produced (a) DO utterances and (b) PO utterances. Error bars denote Cousineau-Morey-corrected within-subjects 95% confidence intervals.

Eye movements during the response phase are shown in Fig 5, separately for DO and PO utterances. As expected, looks to the right interest area fell steeply at the beginning of this phase because the video had ended and the only visual stimulus was a central green circle. However, the looks increased during the pre-choice interval (*Kate gave the…*) on Equi verb trials where participants produced DO. Mixed model analysis confirmed this observation and revealed a significant interaction between response type and a quadratic trend based on verb bias (Table 3). This interaction was the result of a significant verb bias quadratic trend for DO utterances (Estimate = -.75, S.E. = .23, t(651.60) = -3.32, p < .001) but not PO utterances (Estimate = -.02, S.E. = .21, t(43.90) = -.07, p>.9). These results show that for DO utterances in particular, participants were more likely to look at the right interest area during the pre-choice interval for Equi verbs relative to DO-only/PO-only verbs. The right interest area was the location where the transfer action was completed during the video phase. Therefore, these results suggest that participants might have visually recalled the event during the pre-choice interval especially when producing DO with Equi verbs.

**Table 3:** Mixed model analysis of looks to the right interest area in the pre-choice interval.

	Estimate	S.E.	df	t	p
*Fixed Effects*					
Intercept	-3.17	.31	85.66	-10.13	< .001
Bias linear trend	.07	.19	91.59	.39	.701
Bias quadratic trend	-.80	.24	88.67	-3.36	.001
Response type	-.13	.19	74.69	-.66	.512
Response type * Bias linear trend	-.25	.24	242.45	-1.05	.295
Response type * Bias quadratic trend	.78	.31	118.07	2.52	.013

To explore whether individual variability in visualizing the transfer action was associated with the choice of a DO structure for Equi verbs, we computed the correlation between percentage DO produced with Equi verbs and the relative increase in looks to the right interest area for Equi versus DO-only/PO-only verbs (= Mean empirical-logit-transformed proportion of looks to the right interest area when producing DO with Equi minus the average of the corresponding values for DO-only and PO-only verbs). The two measures were positively correlated (r = .26 CI = [.03 0.47]). In other words, participants who showed fixation patterns suggestive of event visualization were more likely to produce DO structures with Equi verbs.

## Discussion

How does prior statistical experience influence sentence production? Which underlying mechanisms enable speakers to select, on occasion, a dispreferred structure over a more preferred one? We manipulated verb bias in a lab-based training protocol and analyzed the effects on subsequent sentence production. The results revealed effects of verb bias and verb-specific competition, and pointed to a role for different executive functions in making structural choices. Below, we highlight the implications of the results and discuss relevant future directions.

### Effects of prior statistical experience on subsequent production

Choice of the dispreferred DO structure was modulated by statistical properties manipulated during lab-based training. Specifically, there was a linear DO-only > Equi > PO-only trend in the proportions of DO structures produced by the speakers. These results corroborate prior findings of verb bias influencing subsequent language use, in both comprehension and production. They extend the confirmatory effects to spoken sentence production in a natural language, going beyond previous evidence examining written production in a natural language and speech using artificial verbs or structures [1,8,9,10,15,16]. The sentence production task used in the present study was naturalistic and involved free choice. Participants described new videos not seen during training using English dative sentences. They showed an overall preference for PO over DO, echoing the general pattern seen in other dative studies [17]. However, prior statistical experience facilitated choosing the dispreferred structure to some extent: verbs that were associated with DO during training were more likely to be used again in that structure.

The findings corroborate the perspective that language learning is dynamic and lifelong [28,29]. They show that adult language users are capable of updating syntactic statistics pertaining to natural language based on a brief exposure session. Future research is needed to address two important remaining questions. First, whether training effects persist over a longer period than a single experimental session merits additional inquiry. Wells and colleagues have shown that reading time improvements for relative clauses can persist for at least several days after lab-based exposure to those structures [29]. Whether similar long-term effects will be seen during spoken language production remains to be established. Second, the generalizability of training effects has not yet been fully ascertained. Future studies could investigate whether the effects of training extend to communicative contexts that are different from the training context (e.g., a new location and/or interlocutor).

### The role of switching when speaking in a single language

Switching ability correlated with DO production across all verb bias conditions in the analysis using normalized difference scores and with DO-only and PO-only verbs in the analysis using unscaled difference scores. Participants who were better able to switch accurately between task rules in the Number-Letter task tended to produce more DO structures during sentence production. Switching is an executive function that is at least partially separable from other regulatory functions like inhibition [20,21]. This ability has been investigated previously in alternating *between* languages, primarily in naming tasks [18,19]. In that literature, switching ability is generally taken to be relevant for enabling bilinguals to adjust their behavior depending on the current language context. To our knowledge, the present study is among the first to suggest that this ability might also be relevant for choosing between sentence structures within a single language. We investigated switching because a previous study found a correlation between switching and structural choice in an artificial language [9]. The current results extend those findings to natural language production. How exactly might switching play a role in choosing between sentence structures? We see two alternative possibilities that are not necessarily mutually exclusive. First, switching might enable speakers to use different task rules that functionally correspond to different syntactic structures, on consecutive trials. For example, switching from conceptualizing a transfer event as being more about the theme to conceptualizing it as being more about the recipient could enable a speaker to switch from PO on one trial to DO on the next. Second, switching could have a broader and potentially more proactive effect by enabling speakers to adjust to the present communicative context. For example, implicitly or explicity recognizing the prevalence of DO structures during the training session could have led participants to form an overarching goal of producing more DO structures within the experimental context. Better switching ability could have helped some speakers to accomplish this goal more effectively. Here, it is worth noting that switching during the present production task was voluntary (i.e., not externally triggered) and happened under conditions of relatively long intervals between utterances (allowing time for top-down selection of a task rule). Growing research suggests that top-down as well as bottom-up factors can influence voluntary switching in the context of non-linguistic tasks [30]. Future studies could manipulate visual and discourse context, salience of different sentence structures, and temporal characteristics to investigate the extent to which the two kinds of factors influence switching between sentence structures during language production.

### Verb-specific competition during sentence production

The present results revealed three distinguishing patterns for producing DO structures with Equi verbs in particular. First, we observed a significant correlation between participants’ ability to inhibit a competing representation and their likelihood of producing DO structures with Equi verbs, but not other verbs. This pattern is consistent with speakers activating both DO and PO for Equi verbs and then suppressing the PO structure to produce the DO structure some of the time. Second, analysis of pre-choice durations also distinguished DO production with Equi verbs from that with other verbs. Pre-choice durations were longer for Equi than DO-only and PO-only verbs when producing DO utterances but not when producing PO utterances, leading to an interaction. This pattern suggests that PO was the default or preferred choice for Equi verbs and that producing the DO structure required additional time. Finally, eye movement analysis prior to the choice of a DO/PO structure showed that participants looked more to the spatial location where the transfer action was previously completed, especially when they produced DO utterances with Equi verbs. Previous research has suggested that eye scanpaths during imagery might play a functional role in accurately recalling the visual scene [31,32]. Thus, a plausible interpretation of our results is that producing DO under verb-specific competition was associated with reactivation and additional processing of the event being described. Individual differences in such reactivation-like fixation patterns correlated with the likelihood of DO production with Equi verbs. This suggests that event reenactment might be useful for selecting a dispreferred structure under verb-specific competition. Together, these convergent results extend understanding of structural competition during sentence production. They show that sentence production might be influenced by competition between structural options not only at the level of the whole language [23,24] but also more specifically at the level of a given verb. When the same verb is associated strongly with two structural alternatives, activation of the verb might automatically activate both structures and require additional processes for resolving verb-specific competition.

### Clarifications regarding aspects of the results

Before closing, it is worth clarifying three aspects of our results a bit further. First, unlike some previous studies [23,24], we found effects of prior statistical experience on pre-choice durations but not onset latencies. The latter null effect could be due to the fact that utterances in our study had a predictable beginning (*Kate …*). Prior research suggests that utterance planning can vary flexibly according to several factors, including structural and lexical accessibility [33]. In this study, repeated use of the same agent could have led participants to begin the utterance prior to making a structural choice and only select a structure while executing the first part of the utterance, resulting in greater sensitivity of the pre-choice duration measure to competition.

Second, eye movement analyses did not reveal any effects involving verb bias during the video phase that preceded the response phase. This is consistent with two alternative possibilities, namely that syntactic information tied to individual verbs was either not accessed or if accessed, did not influence eye movements at this stage. Aspects of our trial structure support the first possibility. We clearly separated the video and the response phases and instructed participants to speak only after the video ended and the green circle appeared. This design could have led participants to delay utterance planning (and thereby the retrieval of verb-specific information). Future studies could investigate whether verb bias effects emerge earlier during the encoding of visual events if speakers are asked to describe the events near-concurrently with the action.

Finally, we noted in the Introduction that the pattern of correlations between inhibition and DO production with different verbs might contradict some a priori intuitions. Specifically, some readers might have hypothesized that inhibition would be most relevant for producing DO with PO-only verbs. The results of this study (and the previous artificial language study [9]) suggest otherwise, by showing that the correlation with inhibition was reliable for Equi but not PO-only verbs. Furthermore, our analyses of pre-choice utterance durations and eye movements both suggested that additional time and processing was involved in selecting the DO structure for Equi but not PO-only verbs. Together, this pattern of results suggests that DO production with PO-only verbs might have relied on a different mechanism than activating and then inhibiting the PO structure, at least some of the time. The correlational patterns suggest that switching might be one such alternative mechanism. The precise way by which switching influences structural choices remains to be established by future studies. For the present, we would like to emphasize a broader point, namely, that different combinations of underlying mechanisms might be recruited in the service of similar behavioral input for different verbs (see also [9]). These mechanistic differences might not be evident from looking at structural choices alone. For example, Fig 2 shows a comparable proportion of DO produced with Equi and PO-only verbs. A post-hoc pairwise comparison revealed that structural choices in the two conditions were not significantly different (Estimate = -.05, S.E. = .11, z = -0.41, p = 0.68). At the same time, other aspects of our results clearly indicate that there were differences between the two conditions in underlying processes. Future research examining sentence production should therefore consider the possibility that measuring structural choices alone might be mechanistically underinformative, and that differences in executive functions between participant samples could lead to different behavioral outcomes across studies.

### Summary

This study demonstrated: a) that prior statistical experience influences naturalistic sentence production; b) that switching ability, which has been evaluated previously in the context of bilingual production, may also be relevant for monolingual sentence production; c) that verbs associated equally with two alternative structures might engender increased competition that manifests itself in utterance characteristics such as longer durations prior to choosing a dispreferred structure; and (d) that inhibition of the preferred structure and visual recall of the event might be used specifically for production with verbs that generate verb-specific competition. These novel results suggest several interesting avenues for future research. Comparing and contrasting the present results with those for structural alternatives like the locatives (*John loaded the wagon with the produce / John loaded the produce on the wagon*)—which contain different ordering of the noun arguments but possess the same syntactic structure—could help clarify the linguistic stage at which different mechanisms exert their influence. Using more finely graded verb biases (e.g., 0% vs. 33% vs 66% vs 100% DO/PO) could help determine the threshold at which verb-specific competition arises. Finally, further exploration is needed to understand how different executive functions like switching and inhibition might interact to facilitate structural choices.

This study shows that verb bias and verb-specific competition affect sentence production. When faced with a structural choice, speakers might minimize effort by choosing the most frequent and accessible option [2]. However, on occasion, speakers can also override that choice. How exactly and how often different speakers produce dispreferred structures depends on both the statistical properties of the input and on individual variation in executive functioning.
